# Supplementary material for: Metformin Suppresses Cancer Stem Cells through AMPK Activation and Inhibition of Protein Prenylation of the Mevalonate Pathway in Colorectal Cancer
Source: Cancers (Basel). 2020 Sep 8;12(9):2554. doi: 10.3390/cancers12092554 (PMC7563617; doi:10.3390/cancers12092554)
Supplement: Supplementary file 1 [file cancers-12-02554-s001.zip › ###Sup_files/##Cancers_final_figure_sup_revision.pptx]

## Slide 1
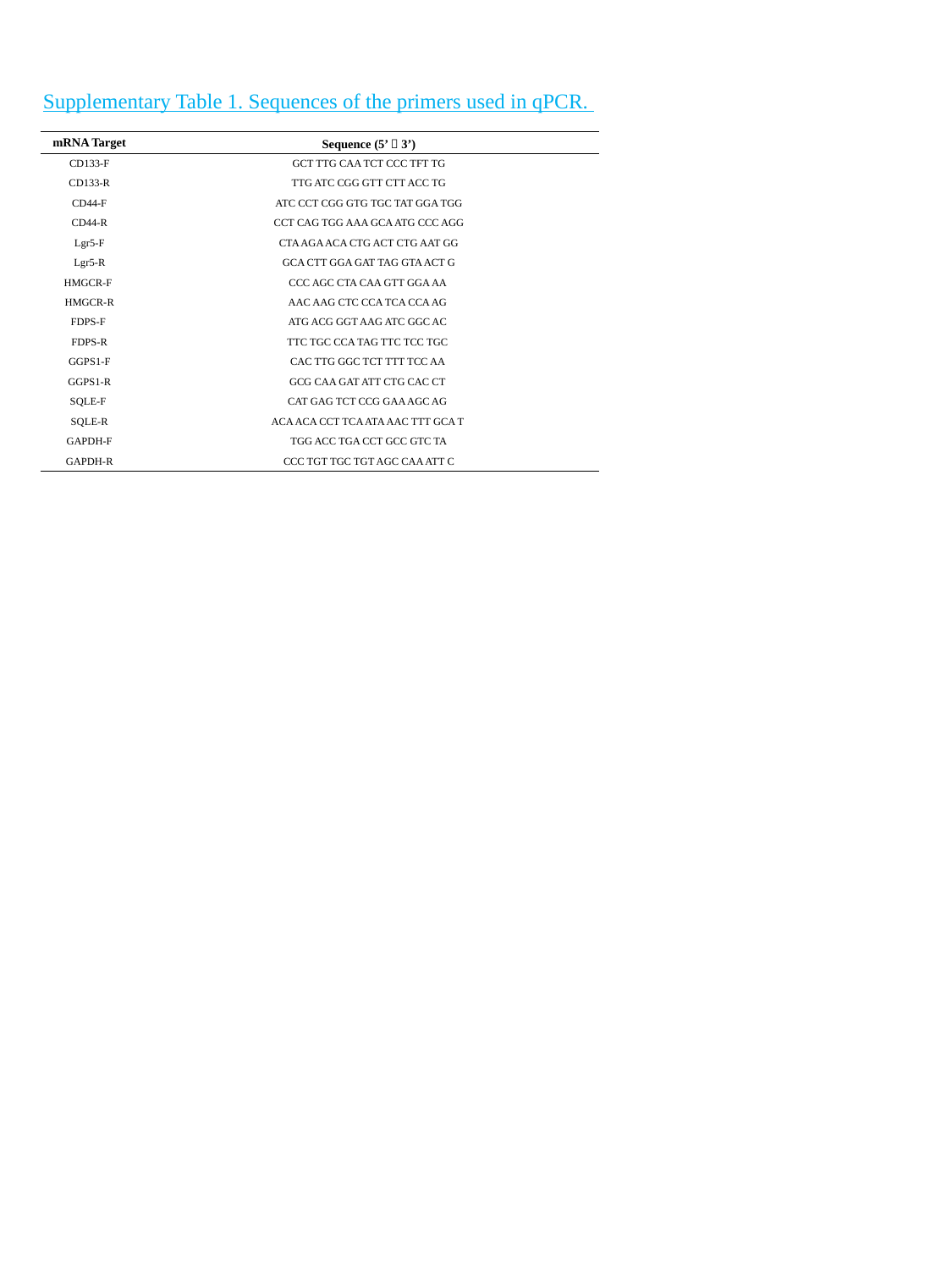

Supplementary Table 1. Sequences of the primers used in qPCR.
| mRNA Target | Sequence (5’  3’) |
| --- | --- |
| CD133-F | GCT TTG CAA TCT CCC TFT TG |
| CD133-R | TTG ATC CGG GTT CTT ACC TG |
| CD44-F | ATC CCT CGG GTG TGC TAT GGA TGG |
| CD44-R | CCT CAG TGG AAA GCA ATG CCC AGG |
| Lgr5-F | CTA AGA ACA CTG ACT CTG AAT GG |
| Lgr5-R | GCA CTT GGA GAT TAG GTA ACT G |
| HMGCR-F | CCC AGC CTA CAA GTT GGA AA |
| HMGCR-R | AAC AAG CTC CCA TCA CCA AG |
| FDPS-F | ATG ACG GGT AAG ATC GGC AC |
| FDPS-R | TTC TGC CCA TAG TTC TCC TGC |
| GGPS1-F | CAC TTG GGC TCT TTT TCC AA |
| GGPS1-R | GCG CAA GAT ATT CTG CAC CT |
| SQLE-F | CAT GAG TCT CCG GAA AGC AG |
| SQLE-R | ACA ACA CCT TCA ATA AAC TTT GCA T |
| GAPDH-F | TGG ACC TGA CCT GCC GTC TA |
| GAPDH-R | CCC TGT TGC TGT AGC CAA ATT C |

## Slide 2
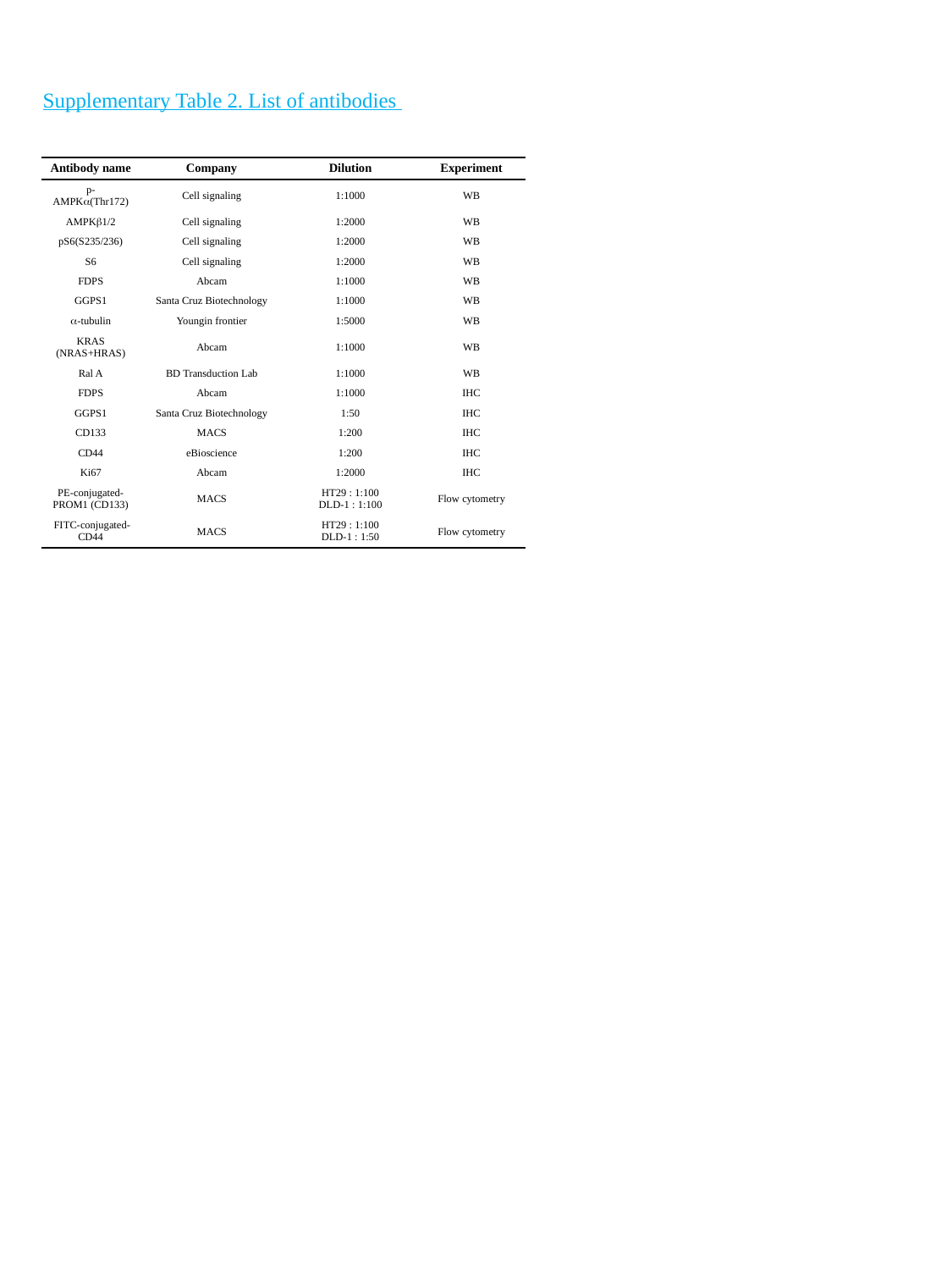

Supplementary Table 2. List of antibodies
| Antibody name | Company | Dilution | Experiment |
| --- | --- | --- | --- |
| p-AMPKa(Thr172) | Cell signaling | 1:1000 | WB |
| AMPKb1/2 | Cell signaling | 1:2000 | WB |
| pS6(S235/236) | Cell signaling | 1:2000 | WB |
| S6 | Cell signaling | 1:2000 | WB |
| FDPS | Abcam | 1:1000 | WB |
| GGPS1 | Santa Cruz Biotechnology | 1:1000 | WB |
| a-tubulin | Youngin frontier | 1:5000 | WB |
| KRAS (NRAS+HRAS) | Abcam | 1:1000 | WB |
| Ral A | BD Transduction Lab | 1:1000 | WB |
| FDPS | Abcam | 1:1000 | IHC |
| GGPS1 | Santa Cruz Biotechnology | 1:50 | IHC |
| CD133 | MACS | 1:200 | IHC |
| CD44 | eBioscience | 1:200 | IHC |
| Ki67 | Abcam | 1:2000 | IHC |
| PE-conjugated-PROM1 (CD133) | MACS | HT29 : 1:100 DLD-1 : 1:100 | Flow cytometry |
| FITC-conjugated-CD44 | MACS | HT29 : 1:100 DLD-1 : 1:50 | Flow cytometry |

## Slide 3
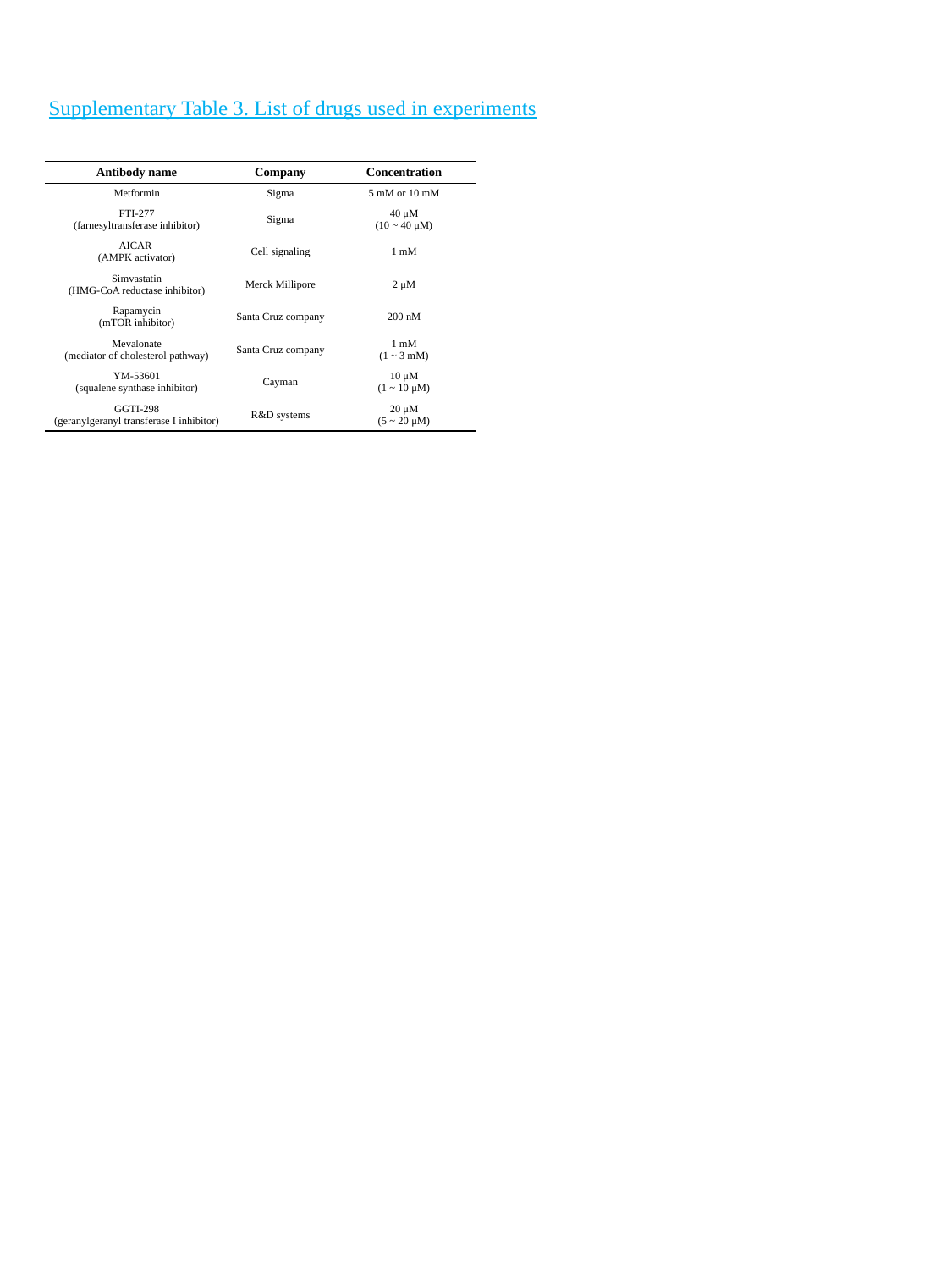

Supplementary Table 3. List of drugs used in experiments
| Antibody name | Company | Concentration |
| --- | --- | --- |
| Metformin | Sigma | 5 mM or 10 mM |
| FTI-277 (farnesyltransferase inhibitor) | Sigma | 40 μM (10 ~ 40 μM) |
| AICAR (AMPK activator) | Cell signaling | 1 mM |
| Simvastatin (HMG-CoA reductase inhibitor) | Merck Millipore | 2 μM |
| Rapamycin (mTOR inhibitor) | Santa Cruz company | 200 nM |
| Mevalonate (mediator of cholesterol pathway) | Santa Cruz company | 1 mM (1 ~ 3 mM) |
| YM-53601 (squalene synthase inhibitor) | Cayman | 10 μM (1 ~ 10 μM) |
| GGTI-298 (geranylgeranyl transferase I inhibitor) | R&D systems | 20 μM (5 ~ 20 μM) |

## Slide 4
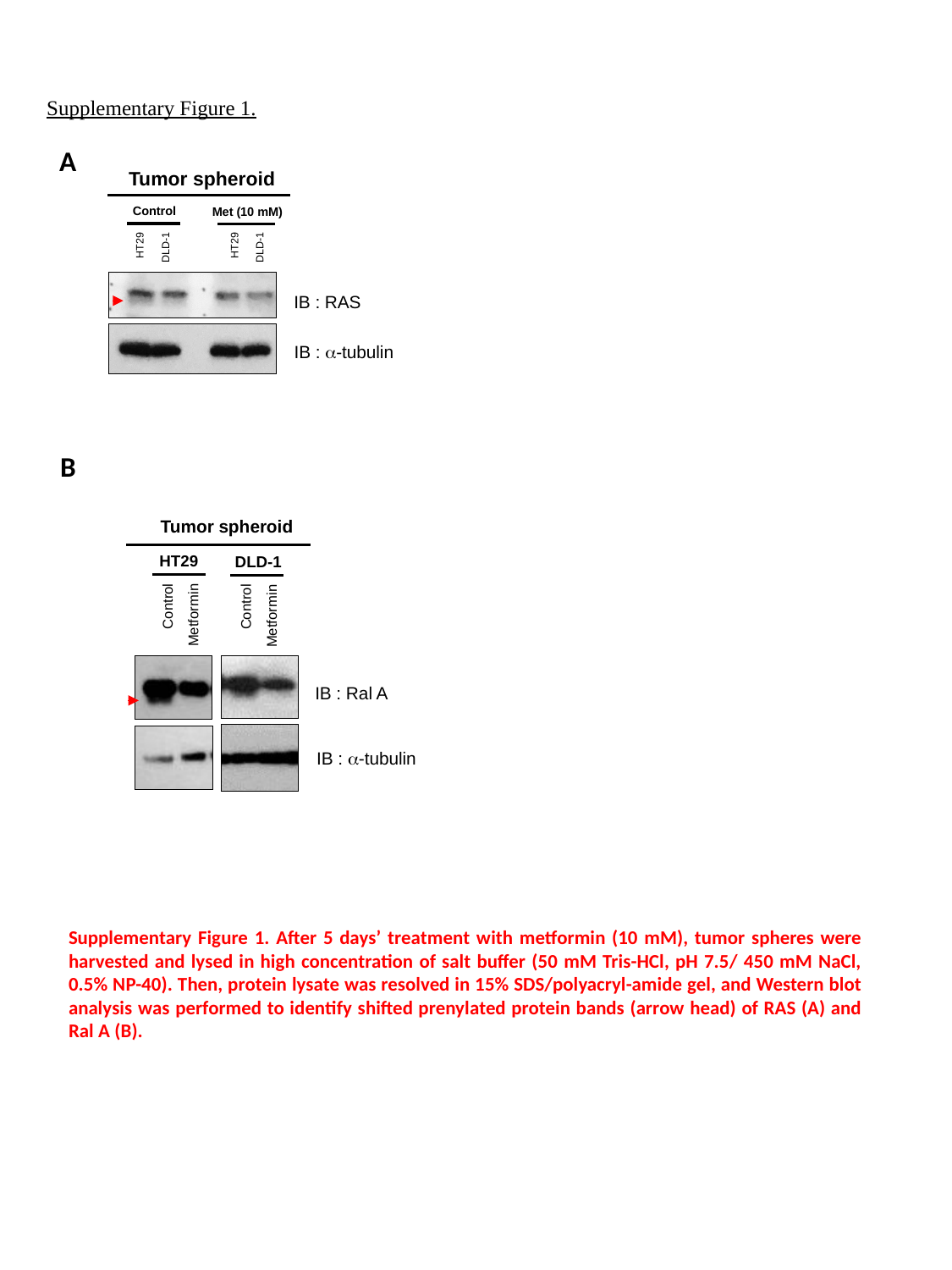

Supplementary Figure 1.
A
Tumor spheroid
Control
Met (10 mM)
HT29
HT29
DLD-1
DLD-1
IB : RAS
IB : a-tubulin
B
Tumor spheroid
HT29
DLD-1
Control
Control
Metformin
Metformin
IB : Ral A
IB : a-tubulin
Supplementary Figure 1. After 5 days’ treatment with metformin (10 mM), tumor spheres were harvested and lysed in high concentration of salt buffer (50 mM Tris-HCl, pH 7.5/ 450 mM NaCl, 0.5% NP-40). Then, protein lysate was resolved in 15% SDS/polyacryl-amide gel, and Western blot analysis was performed to identify shifted prenylated protein bands (arrow head) of RAS (A) and Ral A (B).

## Slide 5
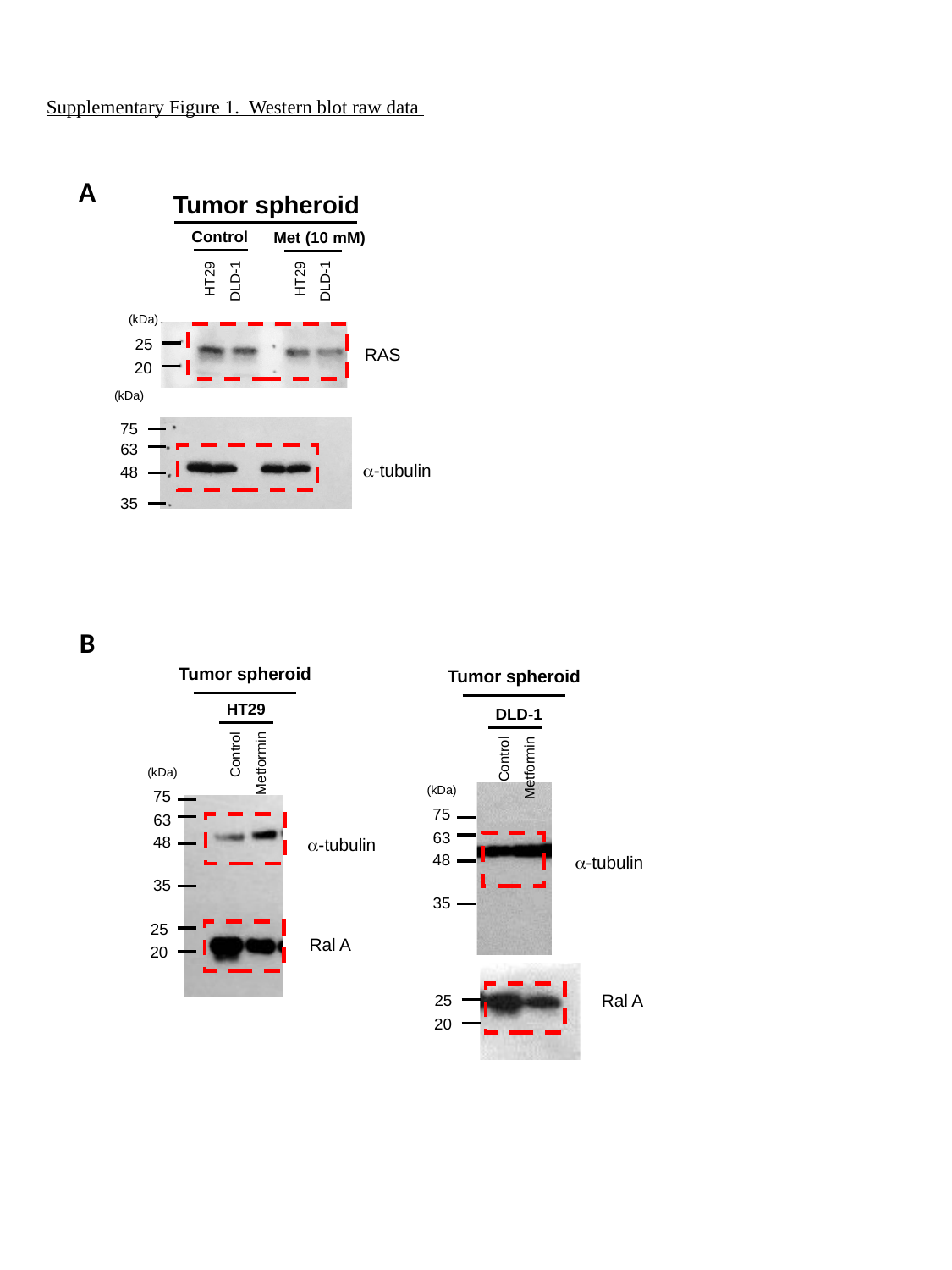

Supplementary Figure 1. Western blot raw data
A
Tumor spheroid
Control
Met (10 mM)
HT29
HT29
DLD-1
DLD-1
(kDa)
25
20
RAS
(kDa)
75
63
a-tubulin
48
35
B
Tumor spheroid
Tumor spheroid
HT29
DLD-1
Control
Control
Metformin
Metformin
(kDa)
(kDa)
75
63
48
35
75
63
48
a-tubulin
a-tubulin
35
25
Ral A
20
Ral A
25
20

## Slide 6
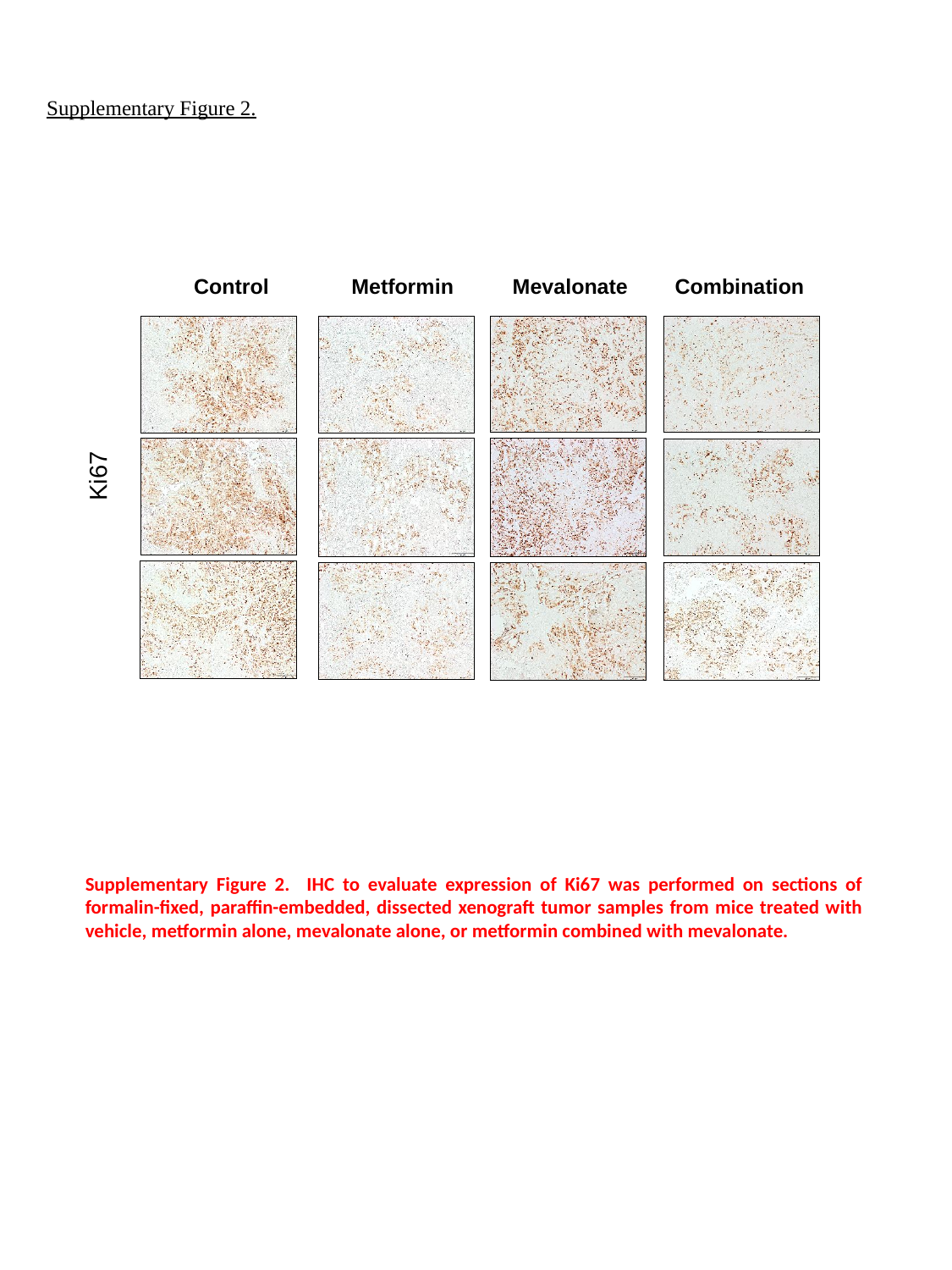

Supplementary Figure 2.
 Control Metformin Mevalonate Combination
Ki67
Supplementary Figure 2. IHC to evaluate expression of Ki67 was performed on sections of formalin-fixed, paraffin-embedded, dissected xenograft tumor samples from mice treated with vehicle, metformin alone, mevalonate alone, or metformin combined with mevalonate.

## Slide 7
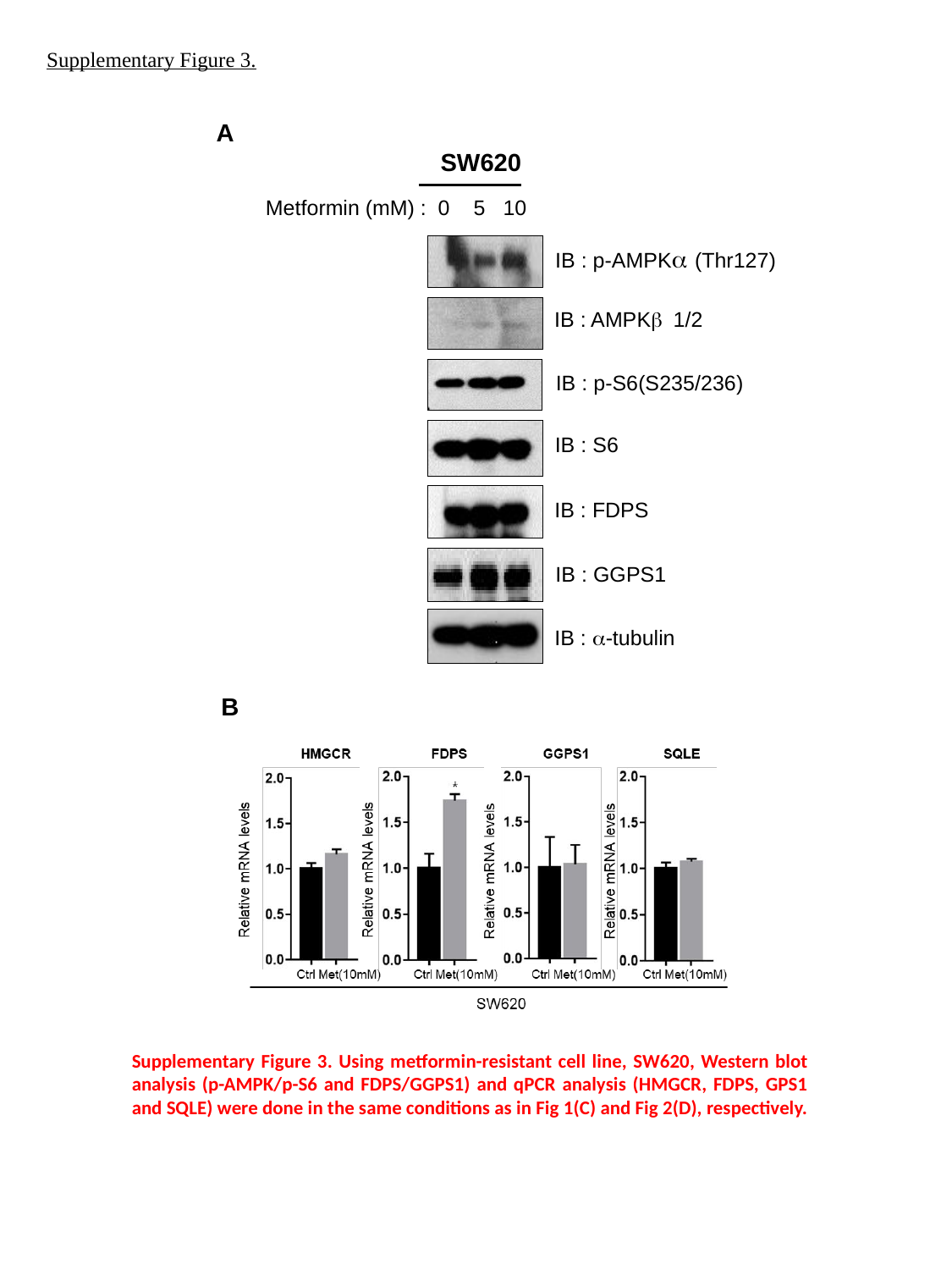

Supplementary Figure 3.
A
SW620
Metformin (mM) : 0 5 10
IB : p-AMPKa (Thr127)
IB : AMPKb 1/2
IB : p-S6(S235/236)
IB : S6
IB : FDPS
IB : GGPS1
IB : a-tubulin
B
Supplementary Figure 3. Using metformin-resistant cell line, SW620, Western blot analysis (p-AMPK/p-S6 and FDPS/GGPS1) and qPCR analysis (HMGCR, FDPS, GPS1 and SQLE) were done in the same conditions as in Fig 1(C) and Fig 2(D), respectively.

## Slide 8
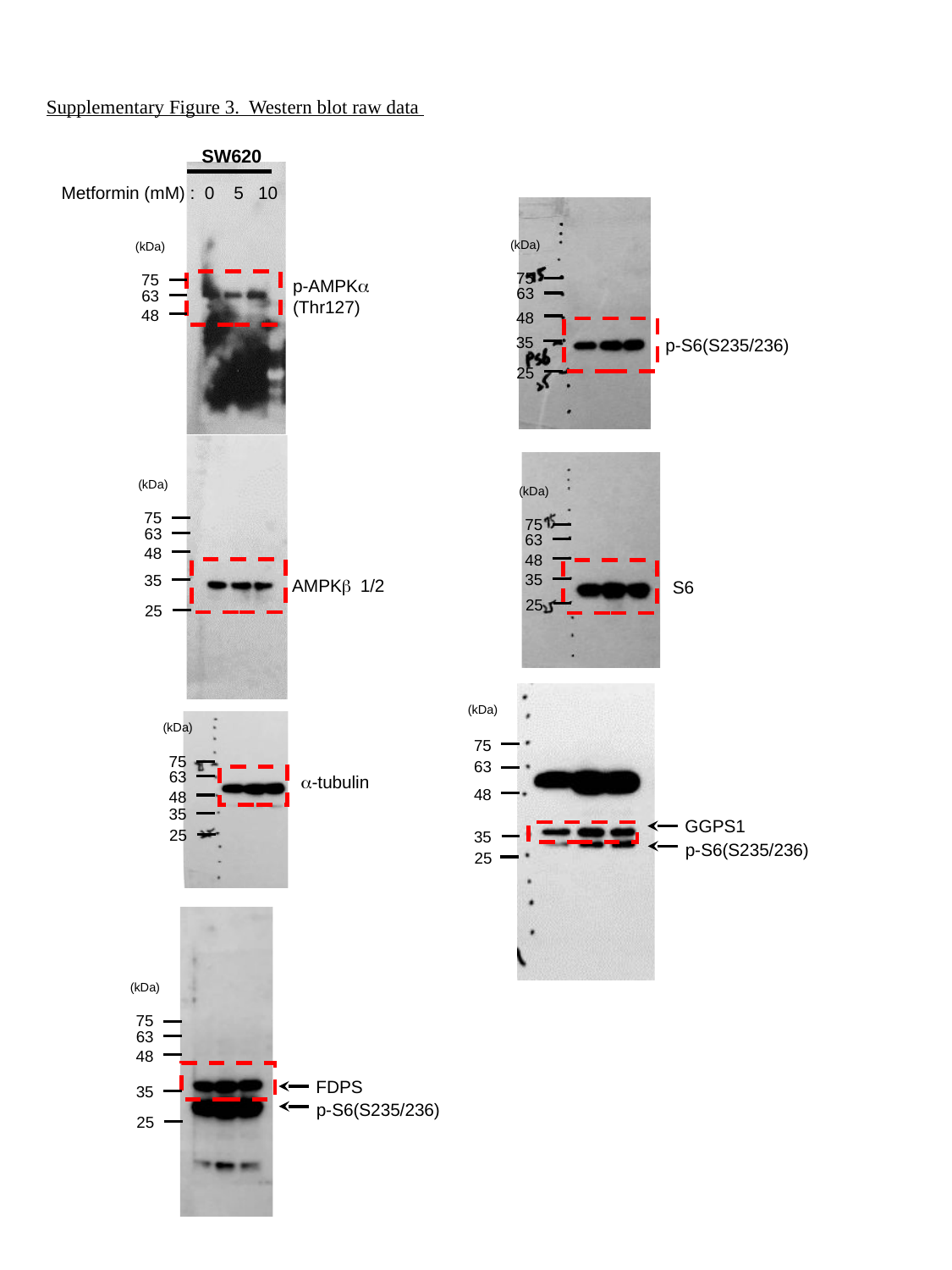

Supplementary Figure 3. Western blot raw data
SW620
Metformin (mM) : 0 5 10
(kDa)
(kDa)
75
75
p-AMPKa (Thr127)
63
63
48
48
35
p-S6(S235/236)
25
(kDa)
(kDa)
75
75
63
63
48
48
35
35
AMPKb 1/2
S6
25
25
(kDa)
(kDa)
75
75
63
63
a-tubulin
48
48
35
GGPS1
25
35
p-S6(S235/236)
25
(kDa)
75
63
48
FDPS
35
p-S6(S235/236)
25
