# Supplementary material for: Metformin Suppresses Cancer Stem Cells through AMPK Activation and Inhibition of Protein Prenylation of the Mevalonate Pathway in Colorectal Cancer
Source: Cancers (Basel). 2020 Sep 8;12(9):2554. doi: 10.3390/cancers12092554 (PMC7563617; doi:10.3390/cancers12092554)
Supplement: Supplementary file 1 [file cancers-12-02554-s001.zip › ###Sup_files/Supplementary_word.docx]

Supplementary Table 1. Sequences of the primers used in qPCR


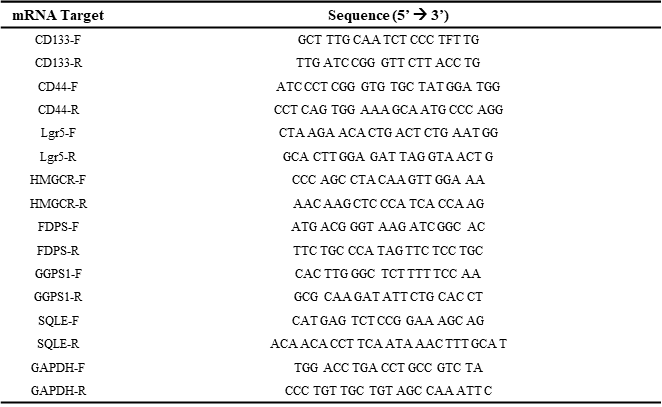


Supplementary Table 2. List of antibodies


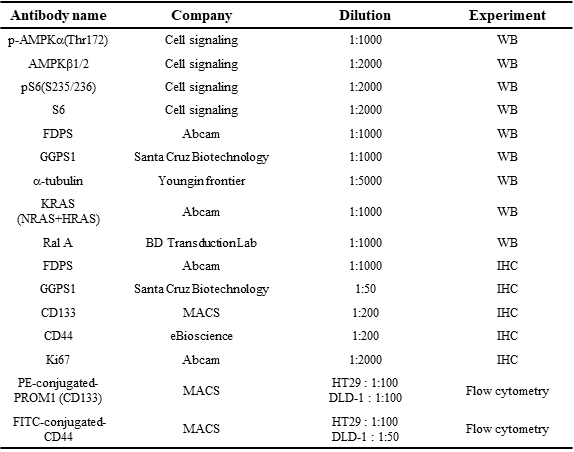


Supplementary Table 3. List of drugs used in experiments


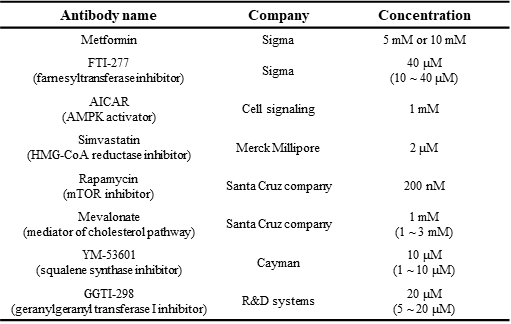


Supplementary Figure 1.

**
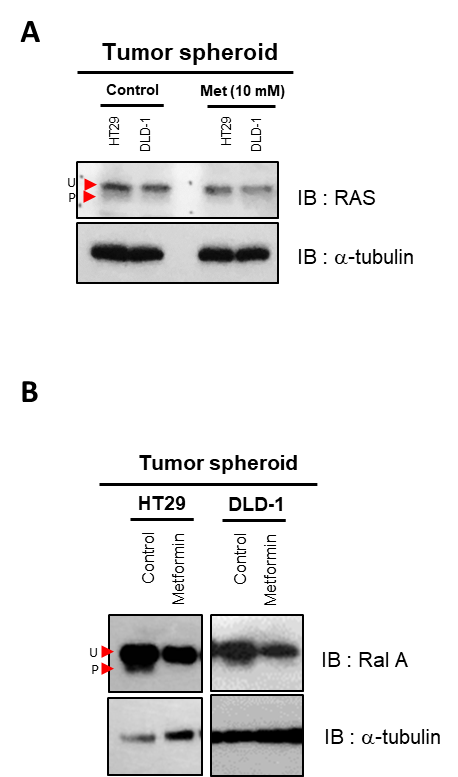
**

**Supplementary Figure 1. After 5 days’ treatment with metformin (10 mM), tumor spheres were harvested and lysed in high concentration of salt buffer (50 mM Tris-HCl, pH 7.5/ 450 mM NaCl, 0.5% NP-40). Then, protein lysate was resolved in 15% SDS/polyacryl-amide gel, and Western blot analysis was performed to identify shifted prenylated protein bands (arrow head) of RAS (A) and Ral A (B).**

Supplementary Figure 1. Western blot raw data

**
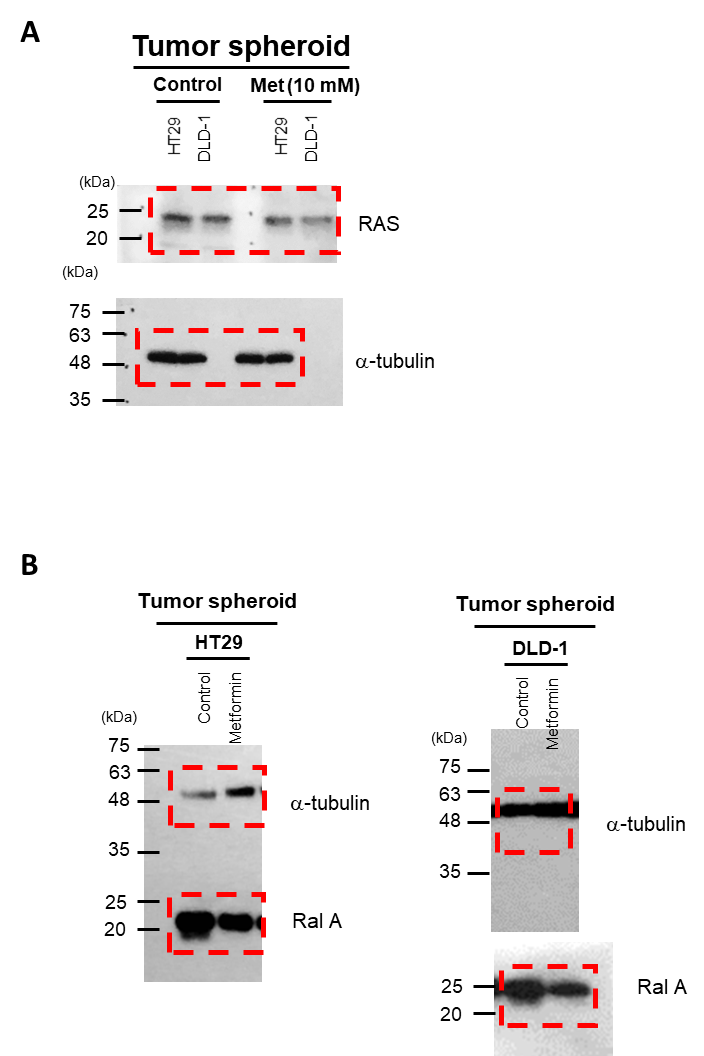
**

Supplementary Figure 2.

**
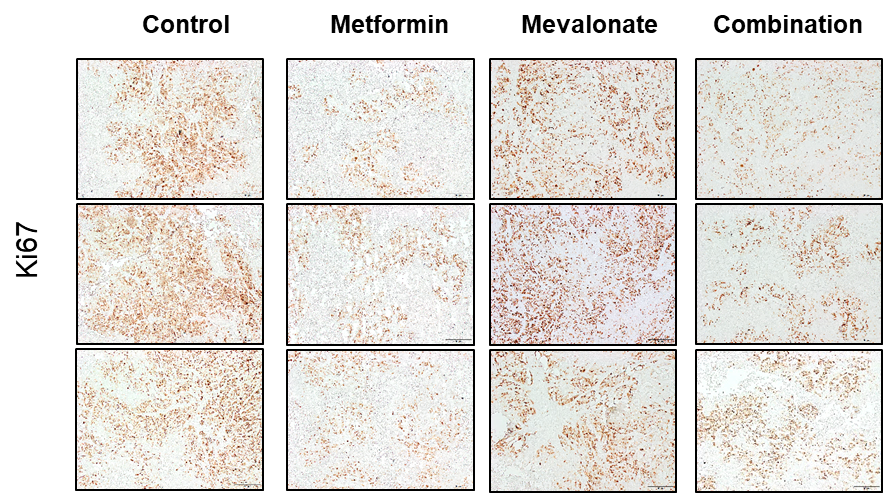
**

**Supplementary Figure 2. IHC to evaluate expression of Ki67 was performed on sections of formalin-fixed, paraffin-embedded, dissected xenograft tumor samples from mice treated with vehicle, metformin alone, mevalonate alone, or metformin combined with mevalonate.**

Supplementary Figure 3.

**
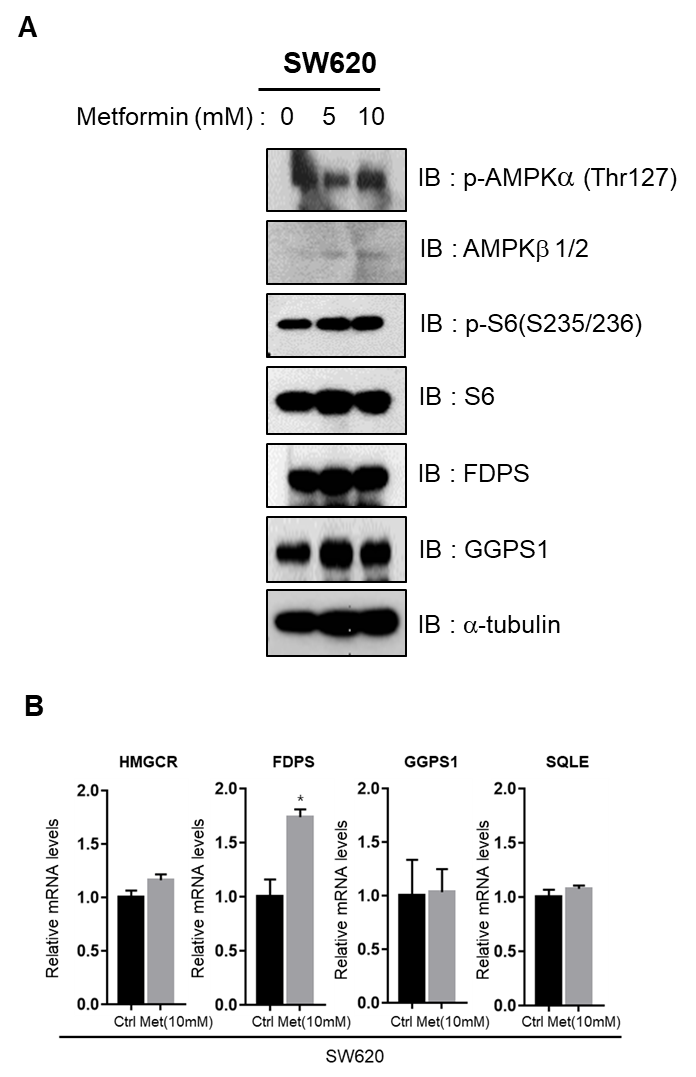
**

**Supplementary Figure 3. Using metformin-resistant cell line, SW620, Western blot analysis (p-AMPK/p-S6 and FDPS/GGPS1) and qPCR analysis (HMGCR, FDPS, GPS1 and SQLE) were done in the same conditions as in Fig 1(C) and Fig 2(D), respectively.**

Supplementary Figure 3. Western blot raw data

**
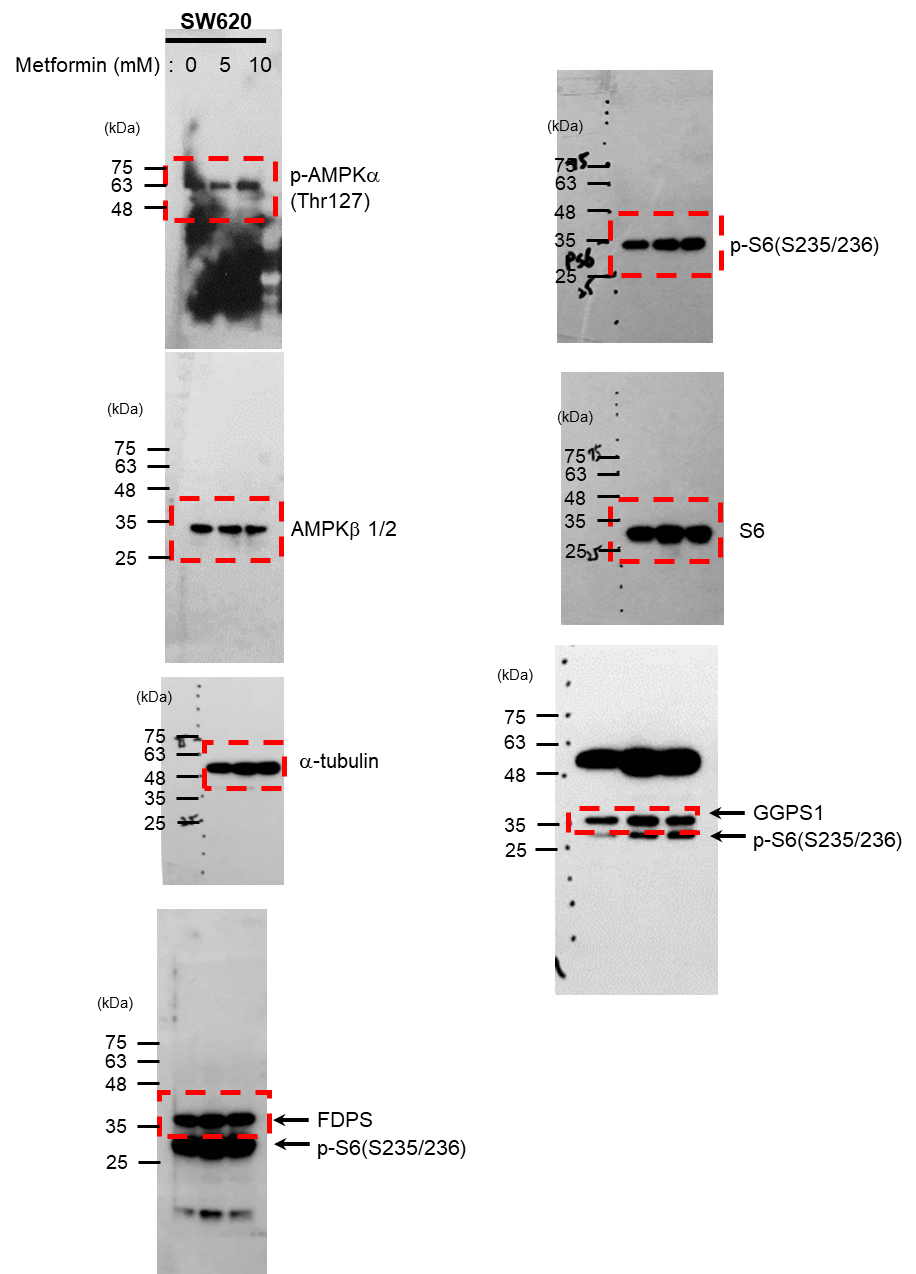
**
