# Supplementary figures and images for: Metformin Suppresses Cancer Stem Cells through AMPK Activation and Inhibition of Protein Prenylation of the Mevalonate Pathway in Colorectal Cancer
Source: Cancers (Basel). 2020 Sep 8;12(9):2554. doi: 10.3390/cancers12092554 (PMC7563617; doi:10.3390/cancers12092554)

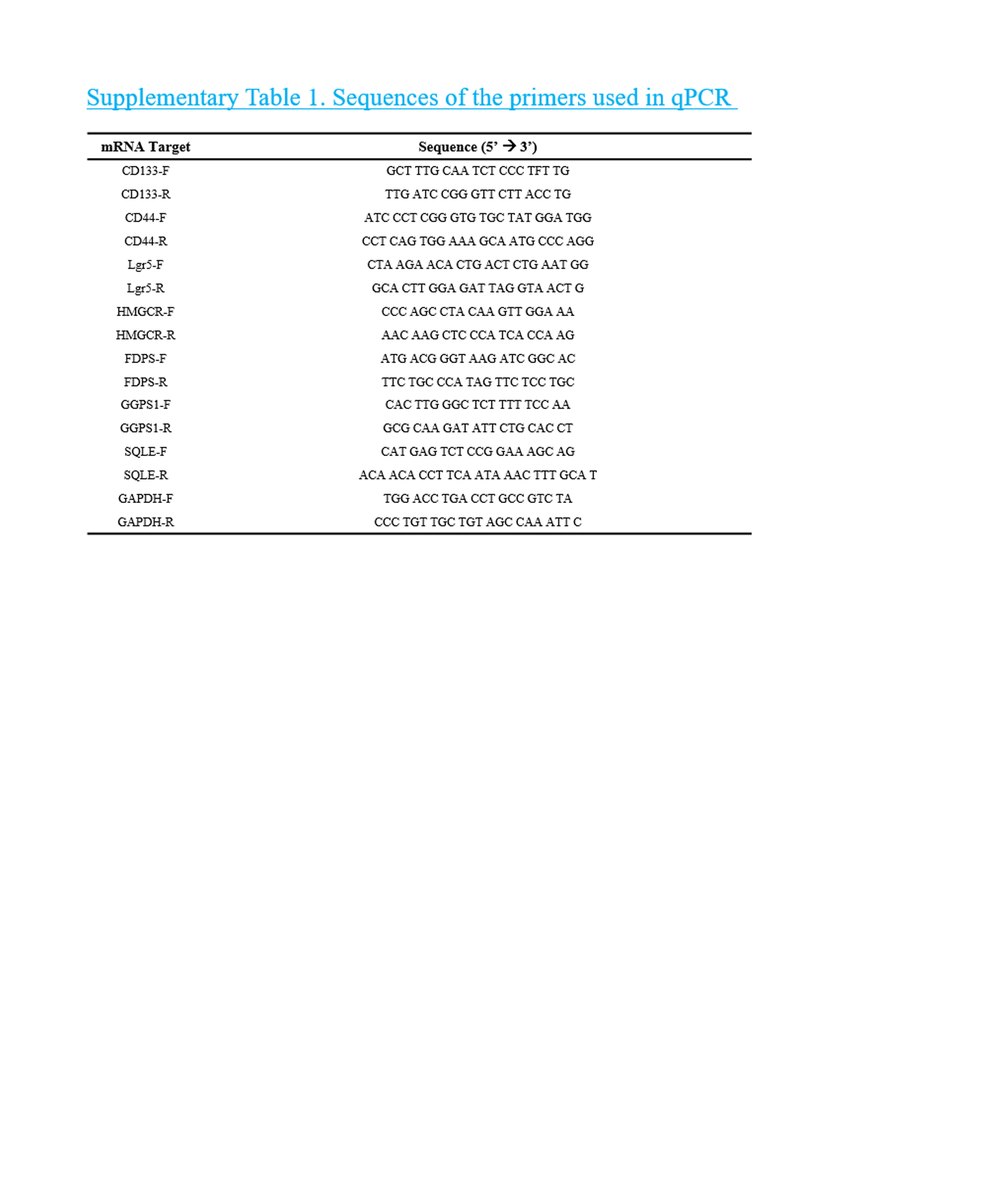

Supplement: Supplementary file 1 [file cancers-12-02554-s001.zip › ###Sup_files/sup_1.tif]

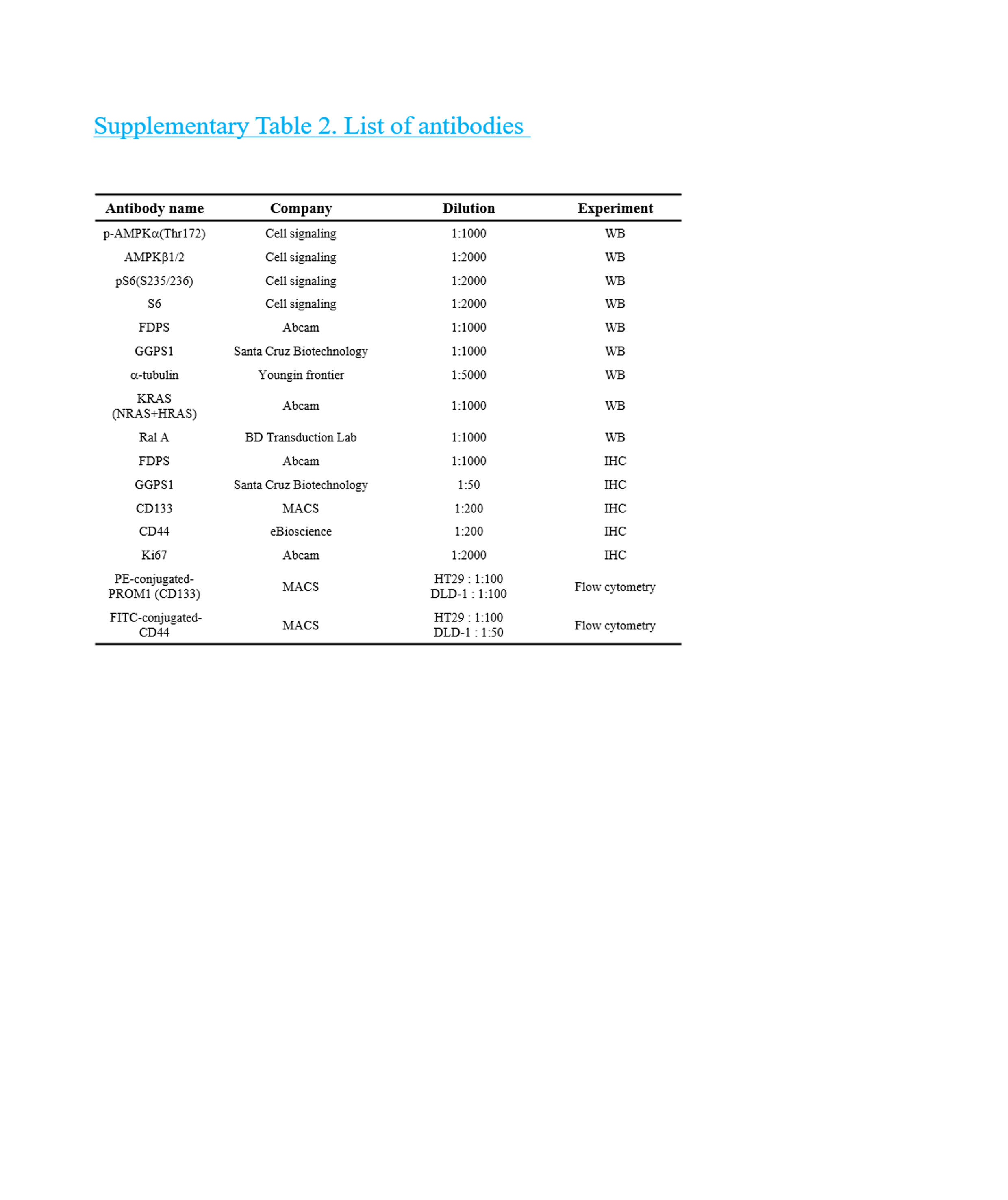

Supplement: Supplementary file 1 [file cancers-12-02554-s001.zip › ###Sup_files/sup_2.tif]

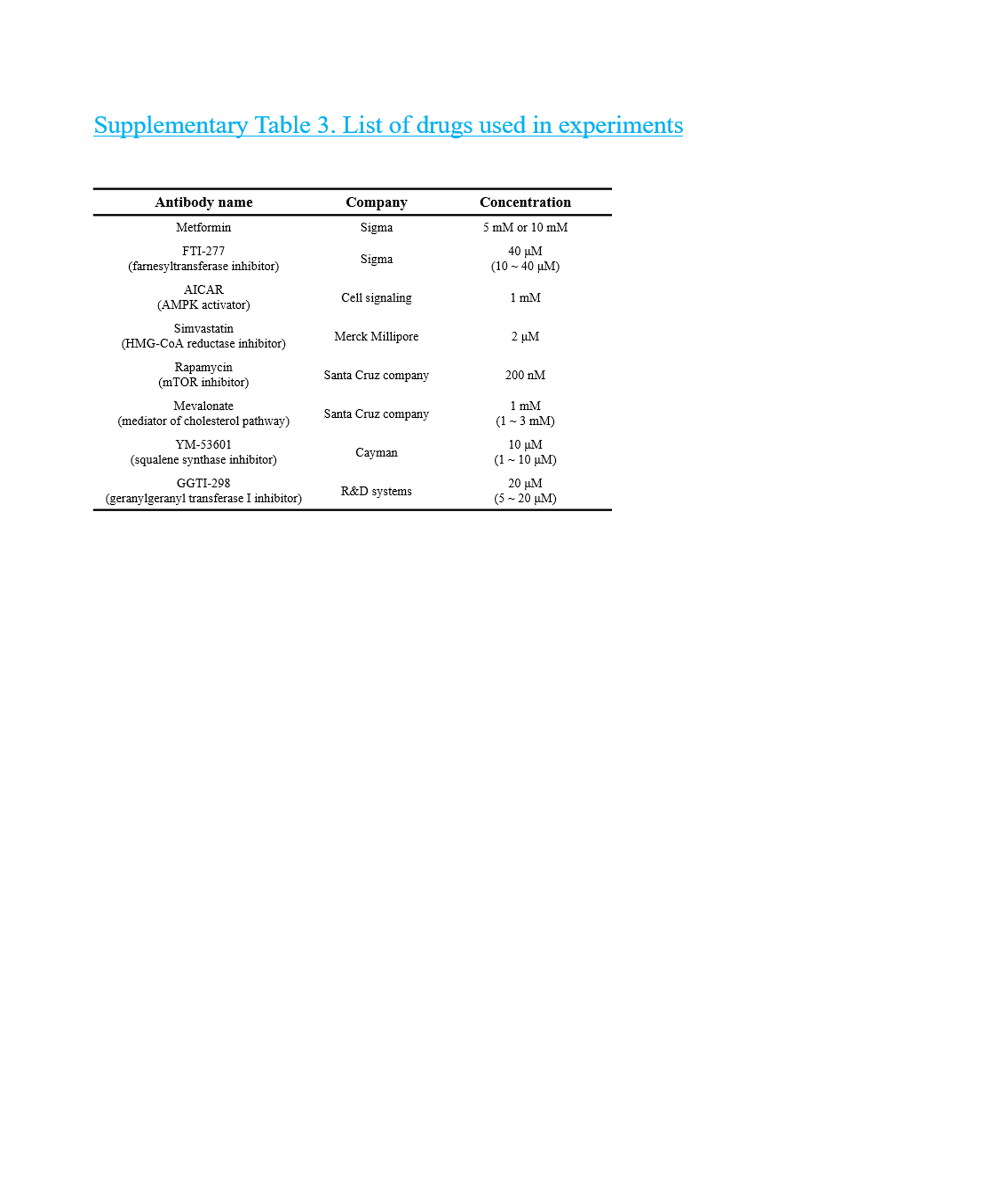

Supplement: Supplementary file 1 [file cancers-12-02554-s001.zip › ###Sup_files/sup_3.tif]

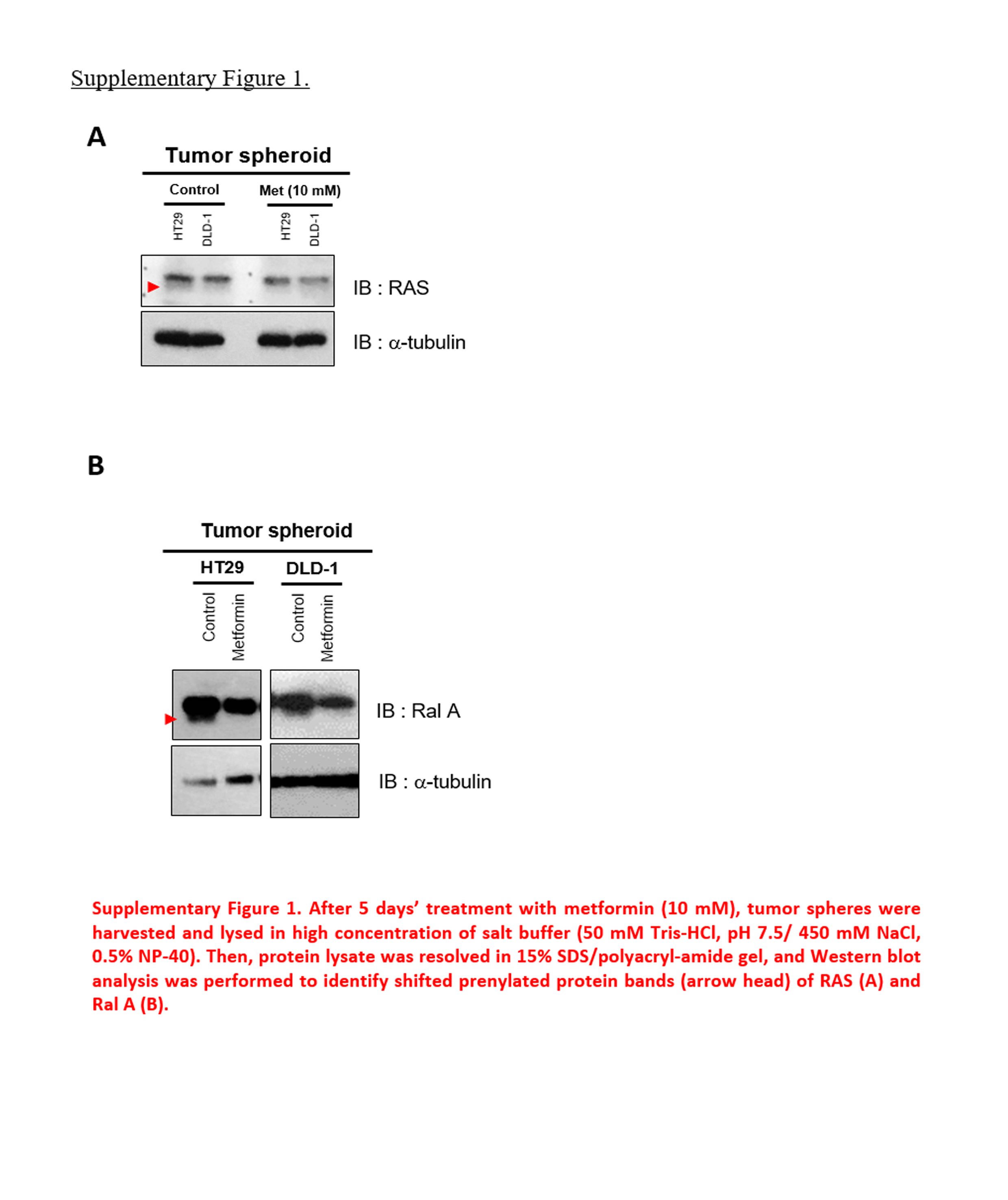

Supplement: Supplementary file 1 [file cancers-12-02554-s001.zip › ###Sup_files/sup_fig_1.tif]

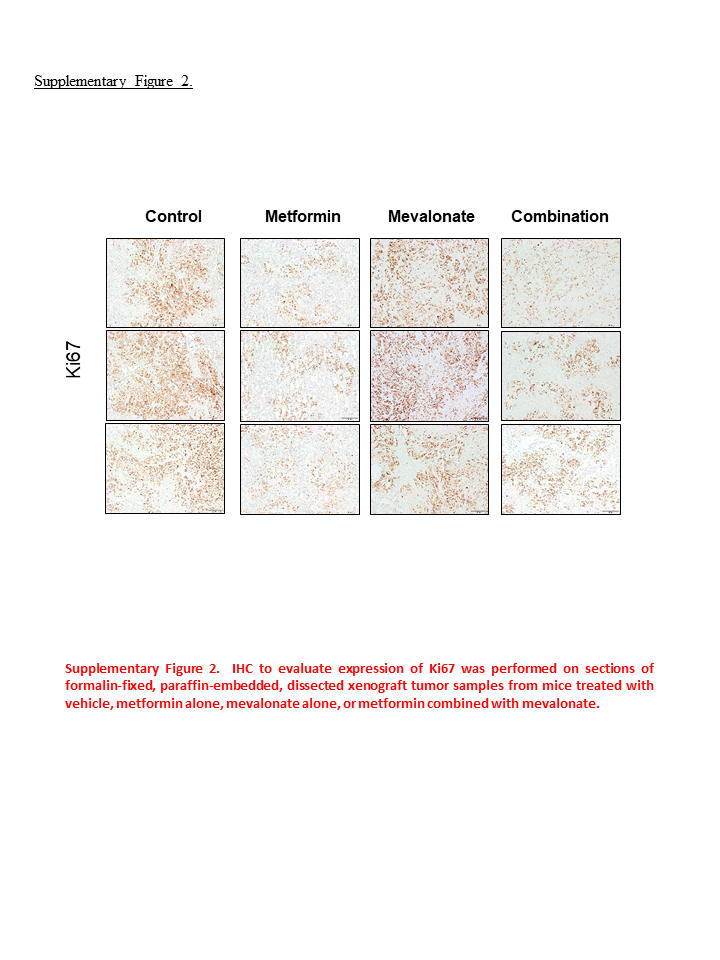

Supplement: Supplementary file 1 [file cancers-12-02554-s001.zip › ###Sup_files/sup_fig_2.tif]

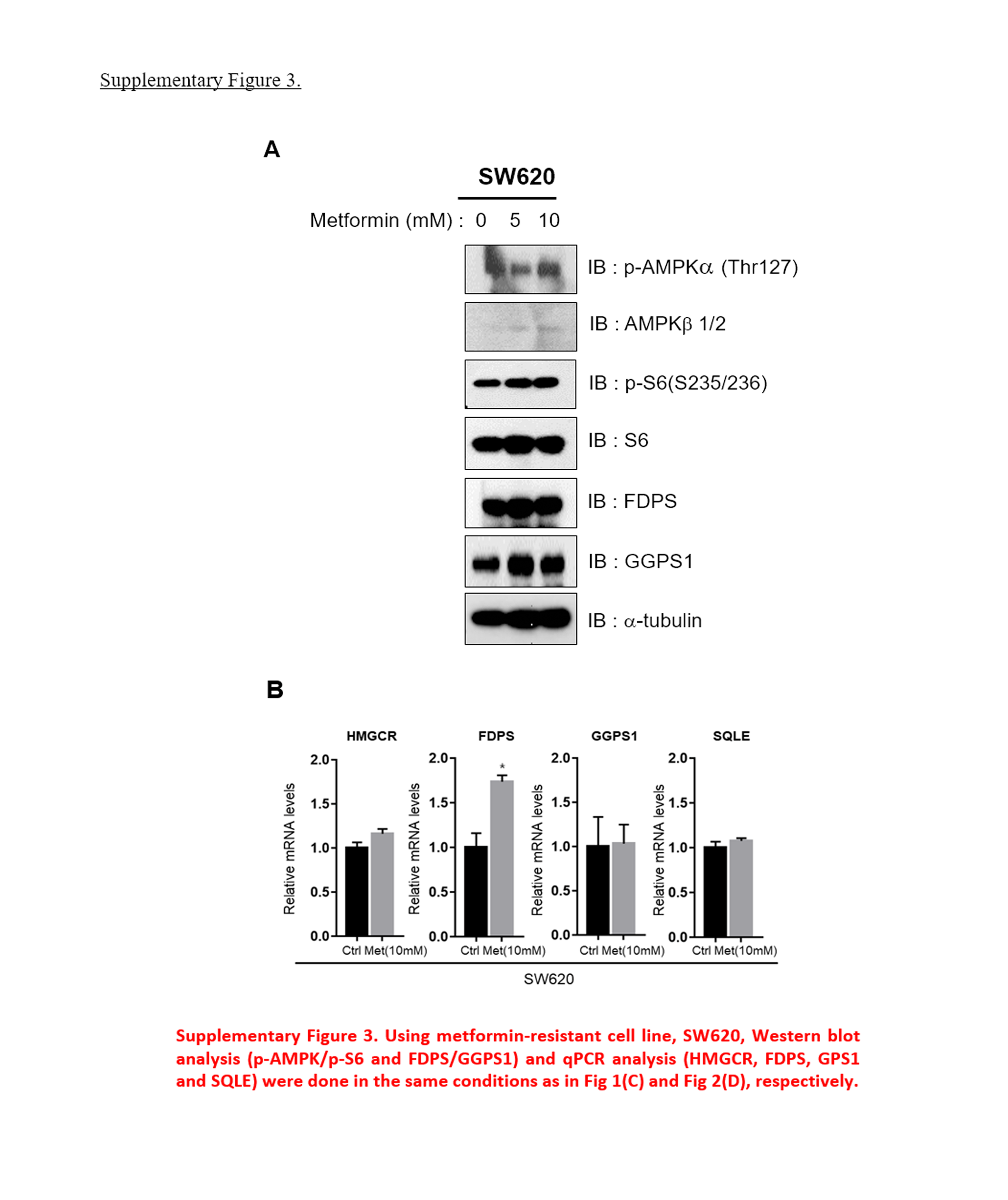

Supplement: Supplementary file 1 [file cancers-12-02554-s001.zip › ###Sup_files/sup_fig_3.tif]

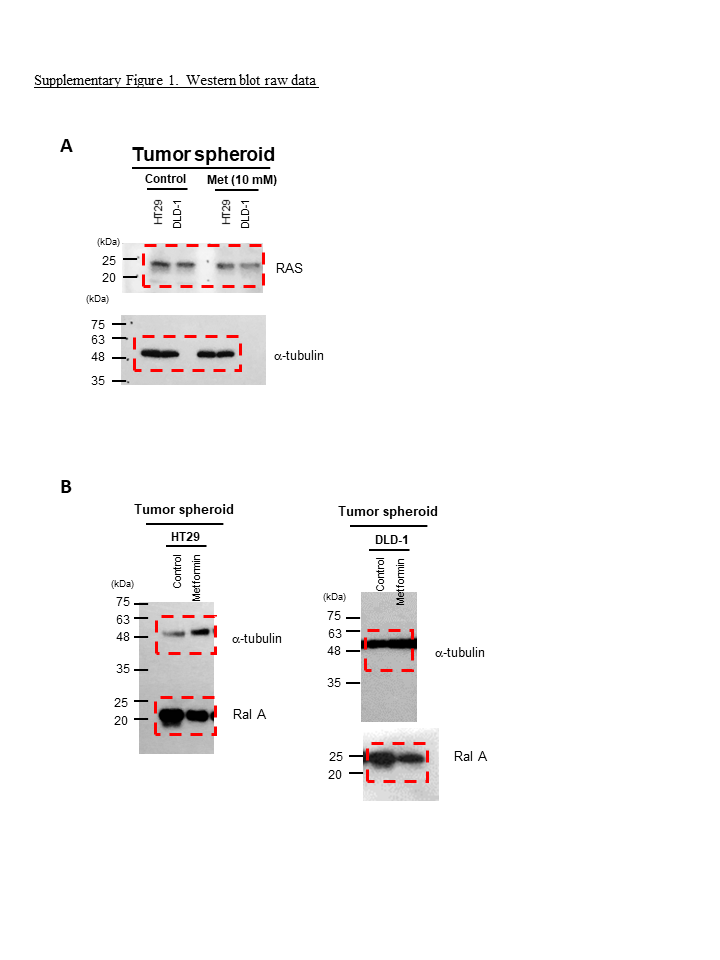

Supplement: Supplementary file 1 [file cancers-12-02554-s001.zip › ###Sup_files/sup_fig_raw_1.tif]

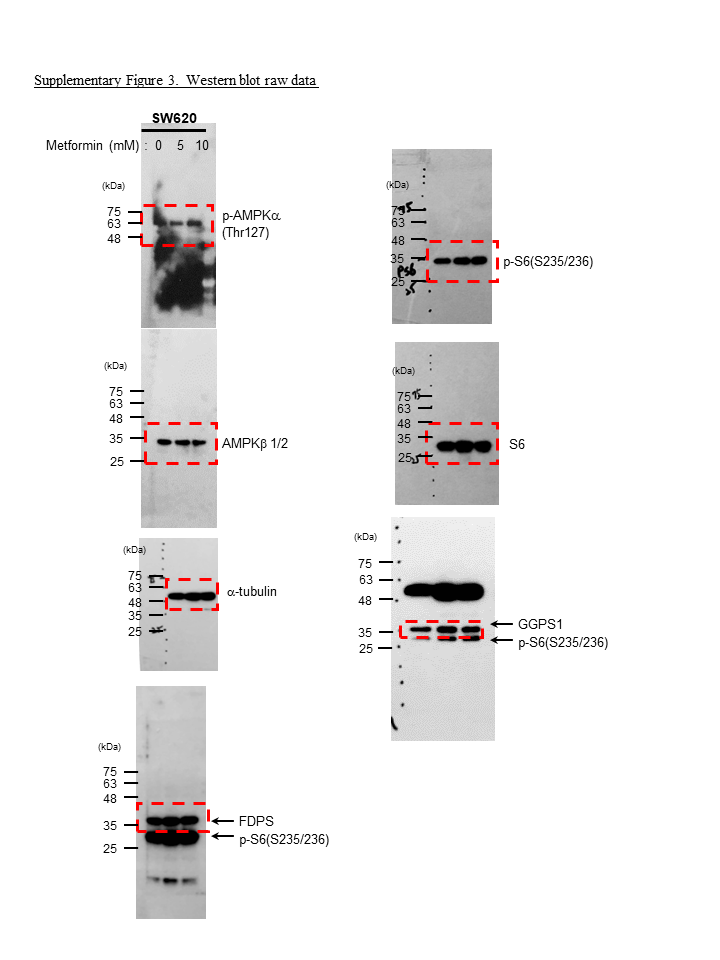

Supplement: Supplementary file 1 [file cancers-12-02554-s001.zip › ###Sup_files/sup_fig_raw_3.tif]
